# Metformin-associated Lactic Acidosis Successfully Treated with Continuous Renal Replacement Therapy

**DOI:** 10.7759/cureus.5330

**Published:** 2019-08-06

**Authors:** Vishal Deepak, Sejal Neel, Abhi Chand Lohana, Armand Tanase

**Affiliations:** 1 Internal Medicine, Western Michigan University Homer Stryker MD School of Medicine, Kalamazoo, USA; 2 Medicine-Pediatrics, Sindh Government Children Hospital, Karachi, PAK; 3 Surgery, Jinnah Sindh Medical University, Karachi, PAK; 4 Critical Care Medicine, Borgess Medical Center, Kalamazoo, USA

**Keywords:** crrt, lactic acidosis, septic shock, metformin, metformin associated lactic acidosis, lactic acidosis in critically ill patient

## Abstract

Metformin-associated lactic acidosis (MALA) is a potentially lethal condition that can result from the use of metformin in the setting of the risk factors such as renal insufficiency or hypoperfusion. We present a case of metformin-associated lactic acidosis incited by pyelonephritis-induced septic shock where use of continuous renal replacement therapy (CRRT) led to good recovery. A 51-year-old female with confusion and abdominal pain was brought to the emergency department (ED). She had a significant past medical history of type ll diabetes mellitus and recurrent urinary tract infections. Prior to the arrival to the hospital, she was conscious but confused and noted to have a low blood glucose level, which was managed with glucose per orally by emergency medical services. While in ED patient was dehydrated and hemodynamically unstable. She failed to respond to intravenous fluids hence vasopressors along with ceftriaxone were initiated. Intubation for mechanical ventilation was performed for respiratory failure and evolving septic shock, sodium bicarbonate for severe metabolic acidosis was started and antibiotics were stepped up to vancomycin and cefepime. The patient was transferred to the medical intensive care unit. Her kidney function continued to worsen, and she remained profoundly acidotic despite aggressive measures. A diagnosis of concomitant MALA was made since vasopressor requirement was less than expected considering the severity of acidosis. Emergent CRRT was initiated, resulting in improvement of acidosis in 24 hours. After she was stabilized vasopressors were stopped, she was extubated, and antibiotics were de-escalated to the oral regimen. MALA is rare but life-threatening complication of metformin use, especially in critically ill patients. CRRT should be considered as the first line in the treatment of metformin-related lactic acidosis, especially in the setting of hemodynamic instability.

## Introduction

Metformin is an oral hypoglycemic agent, recommended as first-line therapy for type 2 diabetes because of its proven safety record, potential cardiovascular benefits, and low cost [1]. It is a biguanide medication that improves the glycemic control in diabetes by decreasing glucose production by the liver and increasing the peripheral sensitivity of insulin [2]. Metformin accounts for one-third of all orally active diabetes drugs prescribed in the United States [3]. Metformin-associated lactic acidosis (MALA) is a rare complication of metformin use, which often develops in the setting of decreased renal excretion of the drug, which may occur during septic shock. However, it can also happen with normal kidney function if excessive amount of the drug is being ingested.

There are no established guidelines regarding the treatment of MALA, due to the rarity of the condition. Different forms of dialysis have been reported to be successful in several case reports. In patients with concomitant septic shock and MALA, continuous renal replacement therapy (CRRT) has been reported to be successful due to hemodynamic instability. However, it has not yet widely been utilized a choice of treatment for MALA. We report a case of MALA masked by septic shock where CRRT lead to rapid recovery. This case was presented orally at Society of Critically Care Medicine conference in February 2019 [4].

## Case presentation

A 51-year-old female with a past medical history of diabetes mellitus type 2 and recurrent urinary tract infections presented to the emergency department (ED) with a chief complaint of abdominal pain and altered mental status. Her medical history was significant for heart failure with reduced ejection fraction (EF 20-25%), chronic atrial fibrillation and pulmonary hypertension. She was on 2000 mg a day of metformin. Two days prior she started feeling unwell and did not eat well. Immediately prior to her admission, due to confusion and low blood glucose levels of 24 mg/dL, emergency medical services were called, and she received 24 g of oral glucose, 25 g of dextrose 50 intravenously and 1 mg of glucagon intramuscularly.

**Figure 1:**
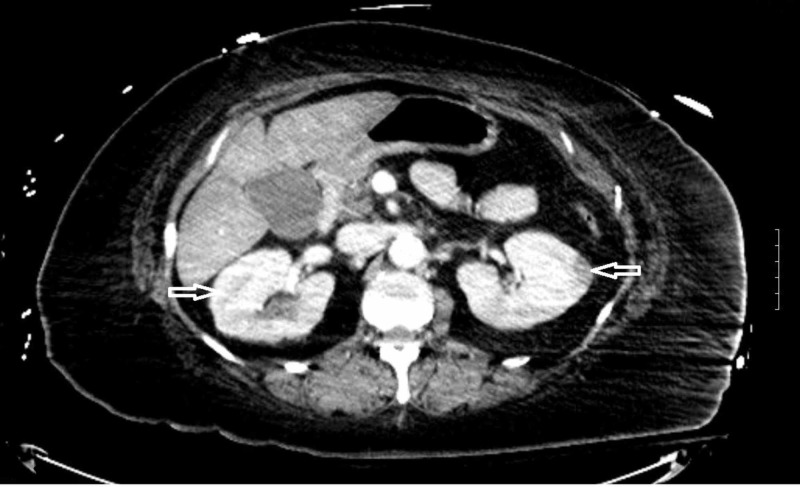
CT scan of the abdomen Cross-sectional CT image of the abdomen at the level of left renal artery with white arrows indicating patchy areas in both the kidneys.

On arrival to ED, the patient was confused and complained of severe abdominal pain in the epigastric region. Vitals were significant for blood pressure: 185/85 mmHg, pulse: 68, temperature: 33.5°C, respiratory rate: 20 breaths/min, and oxygen saturation: 60% on room air. Physical examination revealed tenderness to palpation in the epigastric region, cold and clammy skin. Laboratory studies at the time of presentation are mentioned below (Table 1). Computed tomography of the abdomen was reported as bilateral patchy areas of pyelonephritis in kidneys (Figure 1).

**Table 1:** Laboratory studies at the time of presentation

Laboratory studies at the time of presentation		
TEST	RESULTS	NORMAL RANGE
Arterial pH	6.64	7.35-7.45
Arterial pCO_2_	26 mmHg	34-46 mmHg
Serum bicarbonate	<5 mmol/L	22-32 mmol/L
Serum creatinine	7.4 mg/dL (Baseline 0.6 mg/dL)	0.4-1.2 mg/dL
Serum sodium	139 mmol/L	135-145 mmol/L
Serum potassium	4.3 mmol/L	3.5-5.1 mmol/L
Serum urea nitrogen	74 mg/dL	8-21 mg/dL
Estimated glomerular filtration rate	7 ml/min/1.73	>61 ml/min/1.73
White blood cell count	16.8 K/uL	4.5-11 K/uL
Absolute neutrophil count	12.1 K/uL	1.8-7.7 K/uL
Hemoglobin	14.5 gm/dL	12-15 gm/dL
Platelet count	236 K/uL	150-200 K/uL
Lactic acid	23.8 mmol/L	0.4-1.3 mmol/L
Lipase	53 IU/L	25-78 IU/L
Serum troponin	0.04 ng/mL	0.00-0.04 ng/mL
Prothrombin time	56.2 seconds	10.3-13.8 seconds
International normalized ratio	4.7	N/A
Urine white blood cells	26	0-4
Urine bacteria	Present	N/A
Blood cultures (Two sets)	No growth after five days	N/A

While in the ED, her blood pressure dropped significantly to 57/33 mmHg. Aggressive fluid resuscitation was started, and she received a total of 3.4 liters of lactated ringer solution. She was given ceftriaxone after blood cultures were obtained. Her blood pressure failed to improve with fluid resuscitation; therefore, she was started on norepinephrine and vasopressin for vasopressor support. A central line was placed. She was also intubated for mechanical ventilation in the setting of evolving septic shock and acute encephalopathy. She received four ampules of sodium bicarbonate and was started on sodium bicarbonate with dextrose infusion for severe metabolic acidosis.

**Figure 2:**
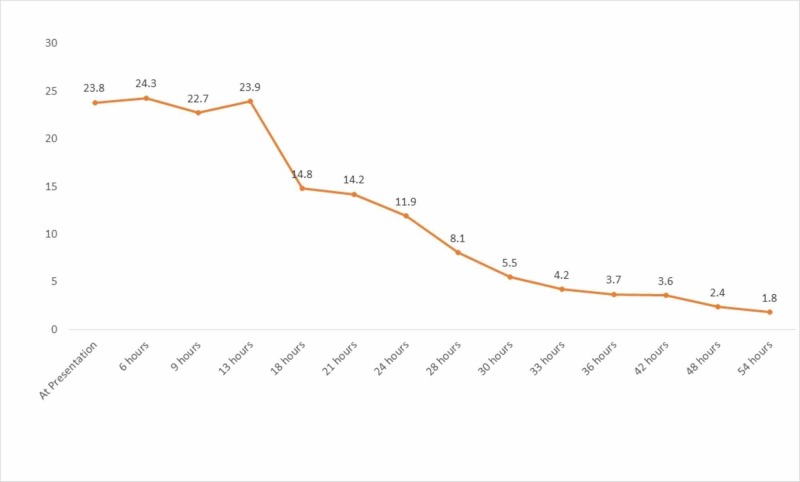
Trend of lactic acid levels Continuous renal replacement therapy (CRRT) was initiated at hour 13th from the time of presentation.

**Figure 3:**
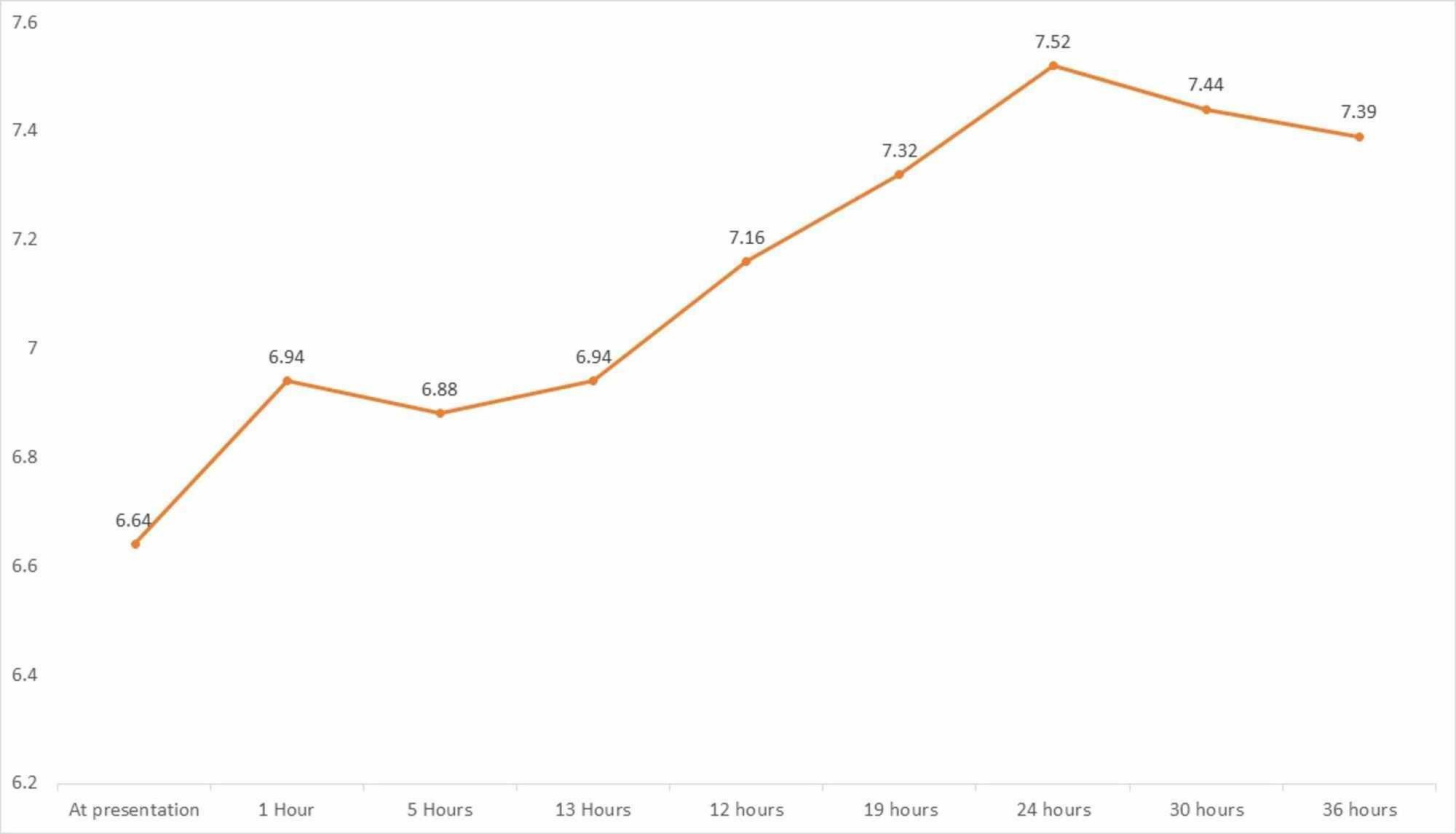
Trend of pH Continuous renal replacement therapy (CRRT) was initiated at hour 13th from the time of presentation.

The patient was transferred to the medical intensive care unit. Antibiotics were escalated to vancomycin and cefepime and intravenous fluids were continued. Over the next eight to 10 hours, her kidney function and lactic acid levels continued to stay significantly deranged despite aggressive measures. While the patient was in obvious septic shock, the amount of vasopressor support was significantly less than expected considering the severity of metabolic acidosis in general and lactic acidosis in specific. The diagnosis of concomitant metformin associated lactic acidosis was made, with pyelonephritis-induced septic shock being the inciting factor. A vascular catheter was placed and emergent CRRT was initiated. A high bicarbonate dialysate (Primasol BGK 4/2.5) was used for CRRT in the setting of severe acidosis. After initiation of CRRT, her pH values returned to normal and lactic acidosis resolved within 24 hours (Figures 2, 3)*. *CRRT was discontinued after four days, with no further requirement of dialysis. She was extubated 30 hours after intubation and vasopressors were stopped within 72 hours as she improved back to baseline. Blood cultures grew no organisms after five days, but urine cultures grew Escherichia coli. Kidney function returned to baseline on day 5, and she was then transferred to the general medicine floor. Antibiotics were de-escalated and continued for a total of seven days.

## Discussion

Metformin has been increasingly prescribed for diabetes mellitus type 2. Use of metformin as a first line anti-diabetic agent has consistently increased from 60% in 2005 to 77% in 2016, with 79% of patients having ever received metformin during that time frame [5]. Metformin-associated lactic acidosis is a rare but life-threatening complication of metformin use, which could become more prevalent because of increasing metformin prescriptions. In the most recent original study estimating the incidence of MALA conducted by Haloob and de Zoysa, the incidence of MALA was actually estimated at 19.46 per 100,000 patient-year exposures to metformin. They also found the relative risk of lactic acidosis in patients taking metformin was 13.53 (95% confidence interval 7.88-21.66) compared with the general population [6].

Lactic acidosis caused by metformin falls into the Type B category of lactic acidosis. While metformin is a rare cause of lactic acidosis, most cases of lactic acidosis are due to marked tissue hypoperfusion resulting from hypovolemia, cardiac failure, sepsis, cardiopulmonary arrest or toxin induced. It has been reported that shock especially of septic etiology is among the most common causes [7].

In the present case, lactic acidosis was caused by metformin accumulation in the setting of acute kidney injury, pyelonephritis and subsequent septic shock. In patients presenting with circulatory collapse, which can also lead to lactic acidosis, the diagnosis of metformin-associated lactic acidosis can be challenging and may potentially be missed. Therefore, a high index of suspicion is needed to make a timely diagnosis.

While conventional hemodialysis remains the ideal choice for management of MALA, it is not suitable in patients with septic shock due to hemodynamic instability. In our case, lactic acidosis was treated with CRRT because of concurrent septic shock. In a retrospective analysis by Mariano et al., survival rate with CRRT in patients with MALA was noted to be 80% [8].

In our case, we focused on treating patient’s profound acidosis rather than removal of drug from the body. Therefore, a dialysate with very high concentration of bicarbonate was used during the CRRT. Suzuki et al. report that the clearance of a drug by CVVH, a form of CRRT, is less than the conventional hemodialysis [9]. However, the benefits of hemodialysis lie more in correcting the metabolic acidosis rather than in clearance of metformin. Renda et al. concluded in their study that accumulation of the drug is less dangerous than other coexisting risk factors for lactic acidosis [10].

Furthermore, in patients with MALA and concurrent septic shock, early correction of acidosis may decrease vasopressor requirements and improve outcomes. Severe acidemia is associated with a high mortality rate and the rapidity of acidemia correction determines patient outcomes [11]. A prospective observational cohort study concluded that non-survivors in the intensive care unit had more profound acidosis as compared to survivors [12].

## Conclusions

This case highlights a comparatively rare, though important complication from metformin - MALA. It is common in critically ill patients and correlates with severity and prognosis. Compared to lactic acidosis of a different origin, metformin-related lactic acidosis is associated with lower mortality and has a better prognosis. Therefore it is very important to make a timely diagnosis. CRRT should be considered as the first line in the treatment of metformin-related lactic acidosis, especially in the setting of hemodynamic instability. From a safety viewpoint, health care providers should enlighten and educate their patients about terminating metformin and other potentially harmful medications in the context of acute illness with volume contraction.
